# Dr. Adel A. F. Mahmoud

**DOI:** 10.4269/ajtmh.18-1941

**Published:** 2018-07-02

**Authors:** Stephanie James, James W. Kazura, Daniel Colley

**Affiliations:** 1 Foundation for NIH, North Bethesda, Maryland;; 2 Center for Global Health & Diseases, Case Western Reserve University, Cleveland, Ohio;; 3 University of Georgia, Center for Tropical and Emerging Global Diseases, Athens, Georgia

The Society mourns the loss of famed physician-scientist and global health leader Dr. Adel Mahmoud, who died June 11, 2018, in New York City as a result of a brain hemorrhage. He is survived by his wife, Dr. Sally Hodder.

Born and raised in Egypt, Dr. Mahmoud received his medical degree from the University of Cairo in 1963. Following a change in political climate, he left Egypt in 1968 to pursue studies in the UK and was awarded a PhD in 1971 from the London School of Hygiene and Tropical Medicine. Committed even at this early stage of his career to the improvement of health in the developing world, he immigrated to the US in 1973 to work as a postdoctoral fellow with the storied Kenneth Warren at Case Western Reserve University, a hub of tropical disease research. Dr. Mahmoud’s early research focused on the role of eosinophils in schistosomiasis, which at the time was a major public health concern in Egypt and remains a significant cause of morbidity in many countries.

Dr. Mahmoud succeeded Dr. Warren as head of the Division of Geographic Medicine at Case Western in 1977, where he continued his research on determinants of infection and disease in human schistosomiasis and nurtured the careers of many of today’s well-known tropical medicine investigators before becoming Chairman of the Department of Medicine in 1987. In 1998, he was recruited to serve as President of Merck Vaccines. In this role, his strong influence on global health continued. At Merck, Dr. Mahmoud played a pivotal role in the development and commercialization of vaccines for rotavirus, human papilloma virus, and shingles, as well as a new quadrivalent formulation of measles-mumps-rubella-varicella vaccine. After retiring from Merck in 2006, he moved to Princeton University as a senior policy analyst at the Woodrow Wilson School of Public and International Affairs. He joined the faculty of Princeton in 2011 as a professor in the Department of Molecular Biology and was an integral member of the university’s Global Health Program.

Dr. Mahmoud served the research and public health community tirelessly. He was a member of the boards of directors of the Global Alliance for Vaccines and Immunizations, the International AIDS Vaccine Initiative, and the International Vaccine Institute. He served on the National Advisory Allergy and Infectious Diseases Council and was chair of the US Delegation of the US-Japan Cooperative Medical Sciences Program. He was called upon to advise the World Health Organization, the National Institutes of Health, the Centers for Disease Control and Prevention, the Rockefeller Foundation, and the National Academies. Throughout his career he received many honors. He was a Fellow of the American College of Physicians and was elected to the National Academy of Medicine, American Society of Clinical Investigation, and American Association of Physicians. He was president of the Central Society for Clinical Research and the International Society of Infectious Diseases. As a decades-long member of ASTMH, Dr. Mahmoud received the Bailey K. Ashford medal in 1983.

Dr. Mahmoud will be remembered for his dynamism, wit, charm, and generosity, his dedication to mentoring young physicians and scientists, his sense of service, and his deep commitment to global public health. Remembrances can be contributed to a blog established by Princeton University to honor his life and legacy: https://blogs.princeton.edu/memorial/2018/06/adel-mahmoud/.

**Figure:**
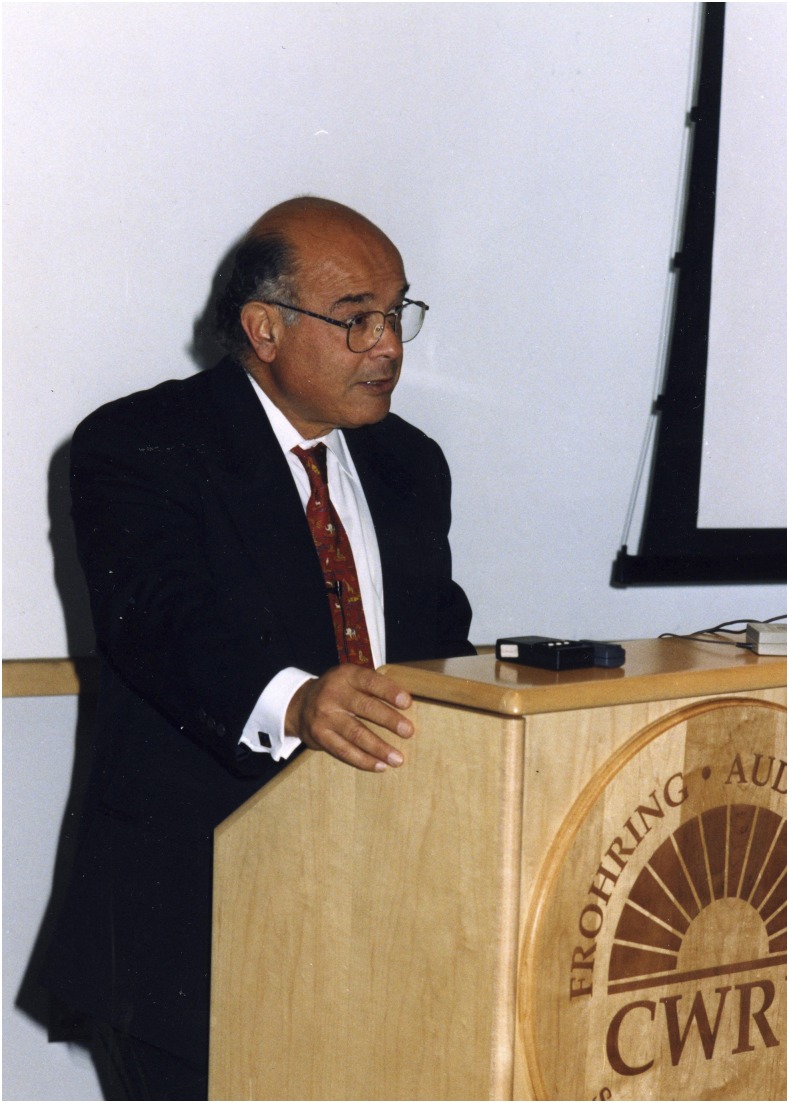
Dr. Adel A. F. Mahmoud

